# Supplementary material for: Activation of a Ductal-to-Endocrine Transdifferentiation Transcriptional Program in the Pancreatic Cancer Cell Line PANC-1 Is Controlled by RAC1 and RAC1b through Antagonistic Regulation of Stemness Factors
Source: Cancers (Basel). 2021 Nov 4;13(21):5541. doi: 10.3390/cancers13215541 (PMC8583136; doi:10.3390/cancers13215541)
Supplement: Supplementary file 1 [file cancers-13-05541-s001.zip › cancers-1409945-SI.pdf]

Figure S1

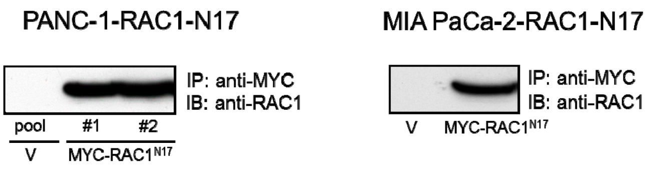

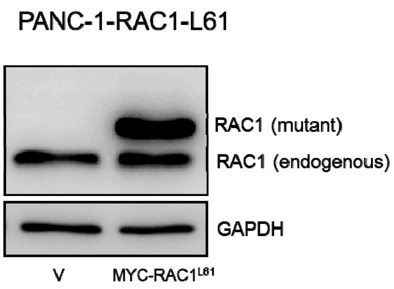

Figure S1. Verification of mutant RAC1 protein expression in transfected PANC-1 and MIA PaCa-2 cells. **(Left-hand panel)** Two individual clones (#1, #2) of PANC-1 cells stably expressing MYC-tagged RAC1^{N17}, and a pool of empty vector (V) control cells were subjected to immunoprecipitation (IP) with anti-MYC antibody followed by immunoblotting (IB) with anti-RAC1 antibody. **(Middle panel)** As in the left panel, except that MIA PaCa-2 cells were used and transfection of MYC-tagged RAC1^{N17} was transient. **(Right-hand panel)** IB analysis of PANC-1 cells transiently transfected with MYC-RAC1^{L61}. The anti-RAC1 antibody recognises both transfected mutant (upper band) and endogenous wild-type (lower band) RAC1 protein. The blot was reprobed with anti-GAPDH antibody to control for equal loading.

Figure S2

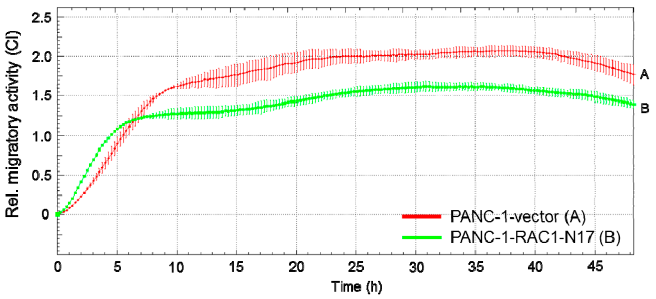

Figure S2. Effect of dominant-negative inhibition of RAC1 activation on migratory activity in PDAC cells. PANC1- cells with stable ectopic expression of RAC1-N17 and empty vector-expressing cells as control were subjected to real-time cell migration assay in normal growth medium. Data represent the mean ± SD of triplicate wells. Differences between cell indices (CI, a dimension-less unit reflecting migratory activities) were first significant ($p < 0.05$) at 8:15 and remained so until completion of the assay.

Figure S3

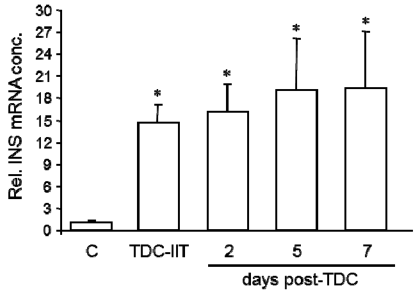

Figure S3. Stability of *INS* expression in PANC-1 cells following TDC with IIT. PANC-1 cells underwent TDC-IIT for 3 days and were subsequently maintained in standard growth medium (without the addition of inflammatory cytokines) for another 7 days. Samples were taken after the indicated times post-TDC and monitored for *INS* expression by qPCR. The data shown (means ± SD of triplicate samples) are representative of three experiments. The asterisks denote significant differences relative to untreated control (C) cells ($p < 0.05$, two-tailed unpaired Student's *t*-test). Differences among the various treated groups were not statistically significant.

Figure S4

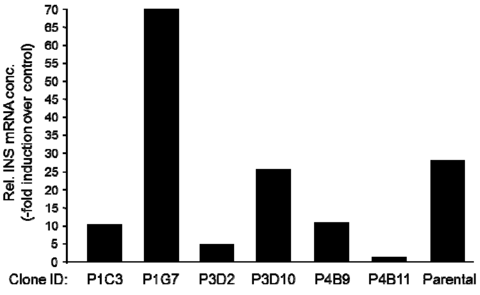

Figure S4. Transcriptional induction of *INS* by IIT based TDC in single cell derived clones of the PANC 1 cell line. Six different individual clones of PANC-1 cells derived by limited dilution were subjected, or not, to TDC with IIT for 3 days and subsequently analysed by qPCR for *INS* mRNA expression. Data are from a representative experiment and are displayed as –fold induction by TDC-IIT over untreated control cells. The standard deviations of the mean values from TDC-IIT and control cells were all below 20%. Parental cells were also shown for comparison.

Figure S5

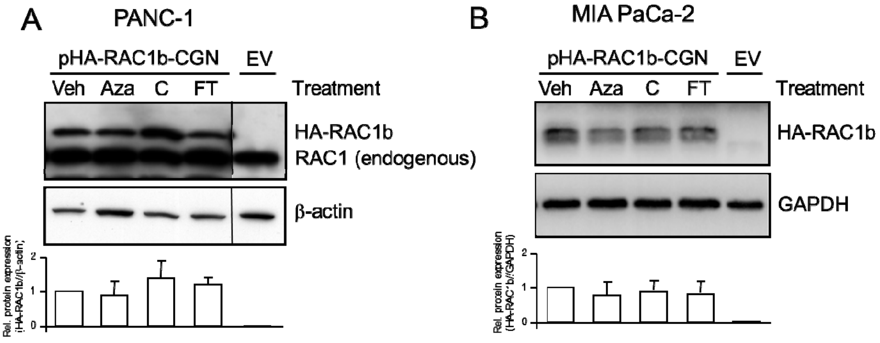

Figure S5. Effect of 5′-Aza or FGFb+transferrin treatment on protein abundance of ectopically expressed HA-RAC1b. **(A)** PANC-1 stably expressing pHA-RAC1b-CGN or empty pCGN vector (EV) were treated for 3 days with growth medium containing either vehicle (Veh) or 1 µM 5′-Aza (Aza) followed by another 3-day incubation in growth medium without Veh or 5′-Aza, or alternatively, for 6 days with either serum-free control medium (C) or serum-free medium supplemented with FGFb+transferrin (FT). Following lysis, cells were processed for immunoblotting of RAC1 with an antibody that recognises both RAC1 and RAC1b. Equal loading was verified with an antibody to β-actin. The thin lines between lanes 4 and 5 indicate removal of irrelevant lanes. **(B)** As in (A), except that MIA PaCa-2 cells were used. Here, an anti-GAPDH antibody was used to control for equal loading. The graphs underneath the blots in (A) and (B) show results from densitometric quantification of band intensities (mean ± SD, n = 3) relative to Veh-treated cells set arbitrarily at 1.0. The changes among the various treatment groups were not statistically significant ($p > 0.05$, Wilcoxon test).

Figure S6

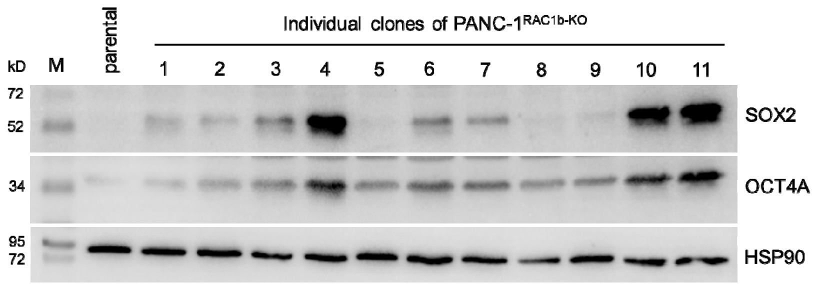

Figure S6. Immunoblot analysis of SOX2 and OCT4A expression in individual clones PANC-1[RAC1b-KO] cells. Eleven different individual (single cell-derived) clones of RAC1b-deficient PANC-1 cells (generated by limited dilution), and wild-type (parental) PANC-1 cells as control, were analysed by immunoblotting for SOX2 and OCT4A. Detection of HSP90 served to verify equal protein loading. Blots shown are representative of three experiments. M, molecular weight marker, kD, kilo Dalton.

Figure S7

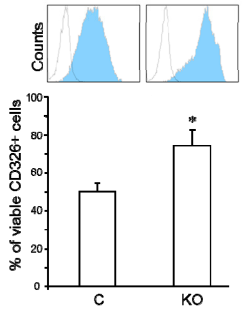

Figure S7. Expression of CD326/EpCAM in RAC1b-deficient PANC-1 cells. PANC1-1[RAC1b-KO] (KO) and control (C) cells were labeled with antibodies to CD326 or isotype control and measured by FACS. Data shown are representative of three independent experiments. Isotype control (white) and anti-CD326 antibody (blue).

Figure S8

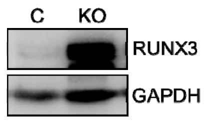

Figure S8. Immunoblot analysis of RUNX3 in PANC-1[RAC1b-KO] cells. PANC1[RAC1b-KO] (KO) and control (C) cells were subjected to immunoblot analysis and sequentially probed with antibodies to RUNX3, and GAPDH as a loading control. The blot shown is representative of three experiments.

Figure S9: Uncropped blots of Figure 4B

Densitometric readings in red color

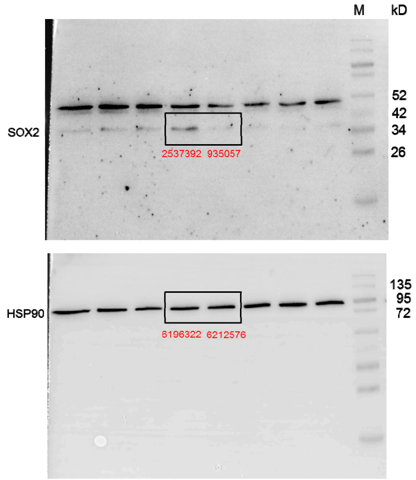

Figure legend: M = molecular weight marker (SM1841, Fermentas/Thermo Fisher Scientific)

Figure S10: Uncropped blots of Figure 4C

Densitometric readings in red color

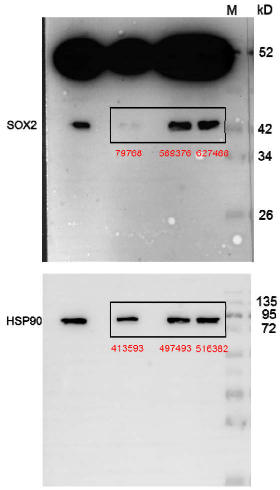

Figure legend: M = molecular weight marker (SM1841, Fermentas/Thermo Fisher Scientific)

Figure S11: Uncropped blots of Figure 5B

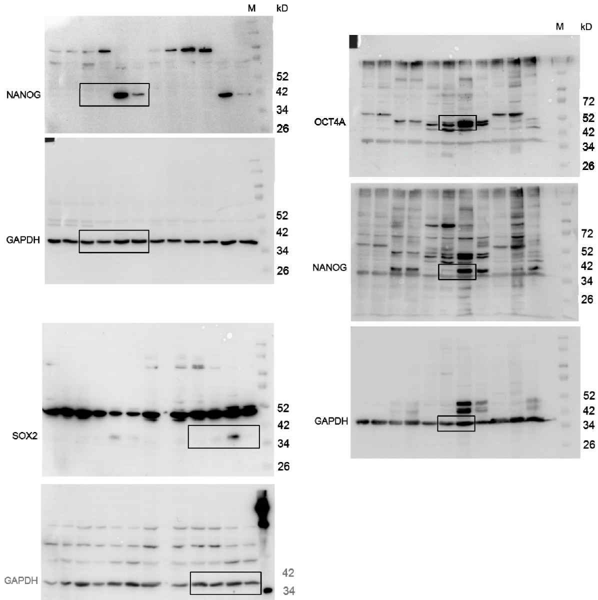

Figure legend: M = molecular weight marker (SM1841, Fermentas/Thermo Fisher Scientific)

Figure S12: Uncropped blots of Figure S1, right-hand panel

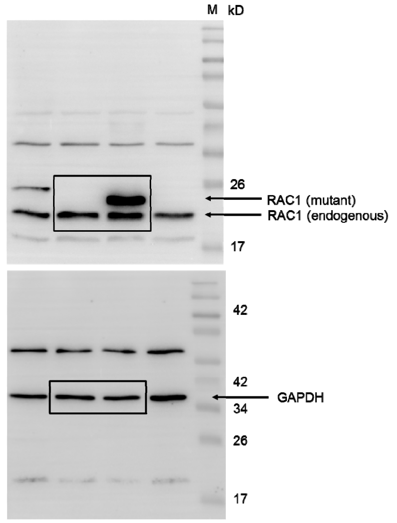

Figure legend: M = molecular weight marker (SM1841, Fermentas/Thermo Fisher Scientific)

Figure S13: Uncropped blots of Figure S2

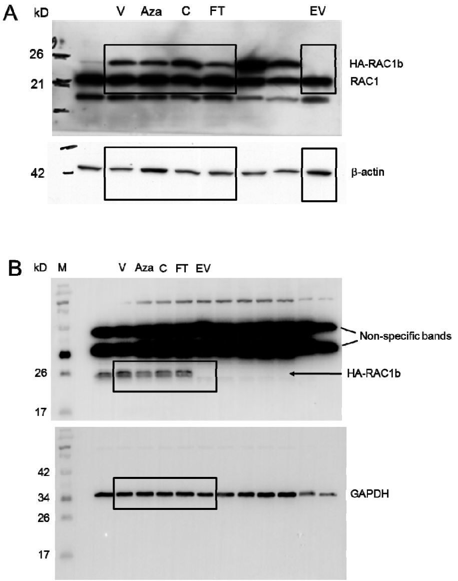

Figure legend: M = molecular weight marker (SM1841, Fermentas/Thermo Fisher Scientific)

Figure S14: Uncropped blots of Figure S6

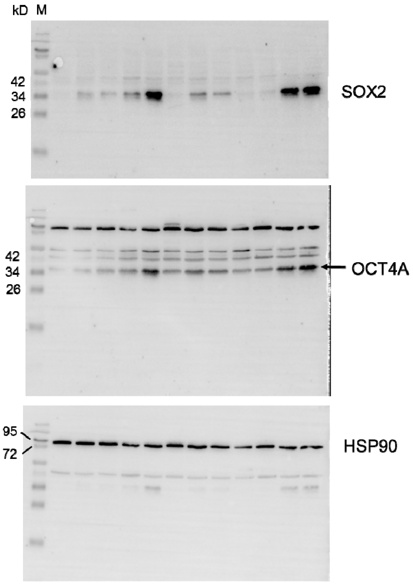

Figure legend: M = molecular weight marker (SM1841, Fermentas/Thermo Fisher Scientific)

Table S1. List of PCR primer sequences

Primer name	Sequence (5’→3’)	GenBank accession
INS-forward	gcagcctttgtgaaccaacac	NM_000207
INS-reverse	ccccgcacactaggtagaga	NM_000207
GAPDH-forward	ttgccatcaatgaccccttca	NM_001357943
GAPDH-reverse	cgcccacttgattttgga	NM_001357943
SLC2A2-forward	ctgcattcagcaattggacctgc	NM_000340
SLC2A2-reverse	tttacacagtctctgtagctcctag	NM_000340
NEUROG3-forward	ggcgctgactcaaacgctgc	NM_020999
NEUROG3-reverse	aagccagactgcctgggctc	NM_020999
KLF4-forward	gccacccacacttgtgatta	NM_004235
KLF4-reverse	cgtcccagtcacagtggtaa	NM_004235
REX1-forward	cagatcctaaacagctcgcag	NM_174900
REX1-reverse	cccgtgtggatgcgcacgt	NM_174900
MAFA-forward	cgagctgaaccggcagctc	NM_201589
MAFA-reverse	gccagcttctcgtatttctccttg	NM_201589
NANOG-forward	gatttgtgggcctgaagaaaact	NM_024865
NANOG-reverse	aggagagacagtctccgtgtgag	NM_024865
POUF5F1-forward	ctctgaggtgtgggggattc	NM_002701
POUF5F1-reverse	ttctggcgccggttacagaacc	NM_002701
TBP-forward	gctggcccatagtgatcttt	NM_003194
TBP-reverse	cttcacacgccaagaaacag	NM_003194
SOX2-forward	agtctccaagcgacgaaaaa	NM_003106
SOX2-reverse	ggaaagttgggatcgaacaa	NM_003106
